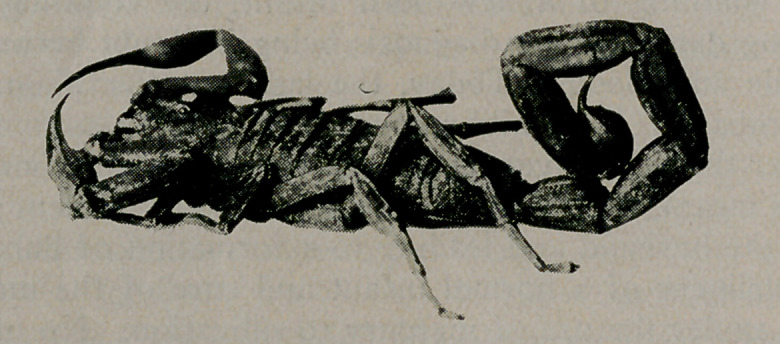# Scorpion Found in Bunch of Bananas

**Published:** 1914-01

**Authors:** 


### ABSTRACTS.

SCORPION FOUND IN BUNCH OF BANANAS. The accompanying cut illustrating a rare but not unknown danger of commerce, is reproduced through the courtesy of *Popular Electricity and the World's Advance,* a journal which is interesting from cover to cover, including its advertisements.

## Figures and Tables

**Figure f1:**